# Impulsivity mediates the association between parenting styles and self-harm in Chinese adolescents

**DOI:** 10.1186/s12889-021-10386-8

**Published:** 2021-02-10

**Authors:** Hailiang Ran, Die Fang, Ahouanse Roland Donald, Rui Wang, Yusan Che, Xingting He, Tianlan Wang, Xiufeng Xu, Jin Lu, Yuanyuan Xiao

**Affiliations:** 1 Department of Epidemiology and Health Statistics, School of Public Health, Kunming Medical University, Kunming, Yunnan China; 2 Psychiatric Department, First Affiliated Hospital of Kunming Medical University, Kunming, Yunnan China; 3 Lincang Psychiatric Hospital, Lincang, Yunnan China; 4 NHC Key Laboratory of Drug Addiction Medicine, The First Affiliated Hospital of Kunming Medical University, Kunming, China

**Keywords:** Impulsivity, Parenting styles, Self-harm, Mediation, Path analysis

## Abstract

**Background:**

Parenting styles are significantly associated with self-harm (SH) in adolescents. Nevertheless, little is known about the mechanism underlying this association. This study primarily aimed to evaluate the potential mediating role of impulsivity in the association between parenting styles and SH in Chinese adolescents.

**Methods:**

Self-administered questionnaires were used to conduct a survey among a sample population consisting of 3146 adolescents in southwest China. Logistic regression analyses were performed to evaluate the association between parenting styles, impulsivity, and SH. A path model investigation further examined the mediating role of impulsivity in terms of the association between parenting styles and SH.

**Results:**

The age range of participants was 10 to 17 years old. The prevalence of SH was 47.0% (95% CI: 36.3–58.0%). Impulsivity, less paternal emotional warmth, maternal over-protection, and rejection were significantly associated with SH. The path model identified impulsivity as a salient mediator, accounting for 23.4% of the total association between parenting styles and SH. The hypothesized path model indicated differences in the parenting styles of fathers and mothers: Impulsivity played a significant mediating role, though only in respect to the maternal over-protection and rejection paths.

**Conclusions:**

For Chinese children and adolescents who experience a harsher maternal parenting style, impulsivity-centered intervention measures might be effective in reducing SH related to parenting styles.

**Supplementary Information:**

The online version contains supplementary material available at 10.1186/s12889-021-10386-8.

## Background

Self-harm (SH), which involves the direct and deliberate destruction of one’s own body regardless of the intention, encompasses behaviors such as scratching or piercing the skin, self-battery, burning, etc. [1]. SH is associated with an elevated risk of various mental health problems and suicidal behaviors [2, 3]. Compared with adulthood, SH behavior is more common during the period of adolescence. In fact, most research studies that focused on examining the influencing factors that contribute to the occurrence, repetition, and severity of SH were carried out among adolescents [4]. Recently, a meta-analysis reported that the overall prevalence of SH was 22.4% among Chinese teenagers [5]. Individual and family negative life events, psychiatric and psychological abnormalities, and sociodemographic factors significantly increase the risk of SH in adolescents [6].

Parenting styles can be understood as a series of psychological constructs that represent standard strategies used by parents in the child-rearing process, which generally include dimensions such as rejection, emotional warmth, and over-protection [7]. Parenting styles are extremely influential in child development. Negative parenting styles, such as rejection and neglect, can increase the risk of mental health issues in children (e.g., depression, anxiety, and hostility) and addictive behaviors [8, 9]. Furthermore, paternal and maternal parenting styles may exert different influences on the mental health and well-being of children, as suggested by recent findings [10]. Some studies revealed that negative parenting styles were also significantly related to SH in young people [11, 12]. In this regard, understanding the developmental path of the association between parenting styles and SH is critical in order to devise intervention measures for SH related to parenting styles.

Impulsivity can manifest in the form of a multitude of behaviors or personality traits, including poor planning skills and persistence, and a predisposition to impulsive actions, especially in the presence of negative emotions [13]. Impulsivity is associated with many risky behaviors among adolescents, including substance and alcohol use, gambling, and unsafe sexual behaviors [14, 15]. In addition, impulsivity is regarded as a possible phenotype of SH [16]. A large body of published research studies highlighted the connection between SH and impulsivity, and generally concluded that individuals with a higher level of impulsivity were more likely to report SH behaviors [17]. As a personality trait, impulsivity is associated with parenting styles [18]. Furthermore, some studies demonstrated that the association between adverse childhood experiences and SH can be mediated by impulsivity [19, 20]. Therefore, as negative parenting styles are, in essence, forms of adverse childhood experience, it is reasonable to theorize that impulsivity may play a mediating role in the association between such experiences and SH. However, to the best of our knowledge, this hypothesis has not been thoroughly investigated.

In the present study, we aimed to explore the association between parenting styles and SH using a large sample population that was representative of Chinese adolescents, and we focused on examining the theorized role of impulsivity as a mediator in regard to this association. We developed the following primary hypotheses: 1) Parenting styles are significantly associated with SH in Chinese adolescents; 2) Impulsivity significantly mediates this association; 3) Differences are observed in impulsivity as a mediator of the association between SH and paternal and maternal parenting styles.

## Materials and methods

### Study design

The present study was inspired by our project entitled “Epidemiological survey on mental health of school children and adolescents in Lincang city”, which focused on understanding the prevalence and associated factors of mental health problems among children and adolescents in southwest China. This survey was carried out in Lingcang city, Yunnan Province, in southwest China from December 1 to December 13, 2019. Considering the representativeness of the sample, we employed a three-stage random cluster sampling strategy: In stage one, Linxiang district was randomly selected from all eight districts and counties within Lincang; in stage two, five primary schools, five junior middle high schools, and four senior middle high schools were randomly selected; finally, 3–4 classes were randomly selected from within each selected school.

Students within the selected classes were excluded if they were: 1) Illiterate; 2) suffered from a severe psychological problem(s); 3) had a physical disorder; 4) had a hearing or communication impairment; or 5) declined to participate. Furthermore, due to the fact that we assessed the presence of suicide ideation and related behavior among the participants of this study, and as it has been suggested that children aged 10 years and older can fully understand the notion of suicide [21], the following inclusion criteria were applied: 1) Aged 10 years old and above, and below 18 years old; 2) Resident in the survey area for at least 6 months per year. Ethical approval of this study was obtained from the Ethics Review Board of Kunming Medical University. Prior to the survey, both the participants and their legal guardians provided their written informed consent. Other details of study design and participants can be found in our previous publication [22].

### Participants

Initially, we identified a total of 3241 adolescents from the 14 schools selected, 88 of whom were further excluded as they did not satisfy the age inclusion criterion of this study. A total of 3146 eligible adolescents were included in the final study, with an effective response rate of 97.3%. For the 3146 participants: the mean age was 13.3 years, with a standard error of 0.6; overall, the number of male students and female students was evenly distributed (1437 versus 1709, respectively); A large proportion of the participants included those of Han ethnicity (67.1%); the most commonly reported education level for both of the parents was “Junior high school and above” (Table 1).

**Table 1:** General features of 3146 adolescents, Lingcang, Yunnan, China, 2019

Features	N (%)	Mean (SE) / Median (IQR)
Demographic		
Sex		
Boys	1437 (45.7)	
Girls	1709 (54.3)	
Age (Mean (SE))		13.32 (0.60)
Ethnicity (%)		
Han	2112 (67.1)	
Other	1034 (32.9)	
Grade		
Primary school	1132 (36.0)	
Junior high school	1069 (34.0)	
Senior high school	945 (30.0)	
Socioeconomic		
Father’s age (Mean (SE))		42.27 (0.51)
Mother’s age (Mean (SE))		39.49 (0.50)
Father’s education level		
Elementary school and below	885 (28.1)	
Junior high school and above	1932 (61.4)	
Missing or unknowns	329 (10.5)	
Mother’s education level		
Elementary school and below	1077 (34.2)	
Junior high school and above	1816 (57.7)	
Missing or Unknowns	253 (8.1)	
Self-harm behavior		
Yes	1480 (47.0)	
No	1666 (53.0)	
Impulsiveness (Median (IQR))		
Combined score		41.67 (10.84)
Motor impulsiveness (Dimension 1)		30 (25)
Assessing impulsive planning (Dimension 2)		47.5 (30)
Cognitive impulsiveness (Dimension 3)		45 (15)
Degree of Impulsivity		
Low (Combined score < 41.67)	1644 (52.25)	
High (Combined score > = 41.67)	1445 (45.93)	
Missing	57 (1.82)	
Parental rearing style (Median (IQR))		
Father		
Rejection		7 (3)
Over-protection		16 (4)
Emotional Warmth		15 (8)
Mother		
Rejection		8 (3)
Over-protection		17 (5)
Emotional Warmth		14 (7)

### Measurements

Data were collected from all participants using a self-administered questionnaire. After participants provided their written informed consent, all participants completed the questionnaire within about 40 min. In order to improve the quality of the survey, each questionnaire was carefully and immediately reviewed by on-site pre-trained personnel who were either undergraduates recruited from a local university or postgraduates with a background of psychiatry or public health from Kunming Medical University. The structured questionnaire contained several parts which measured general characteristics, parenting styles, SH behaviors, impulsivity, mobile phone use, suicide ideation and behavior, resilience, depression, and anxiety. Further details regarding this questionnaire can be obtained by referring to our previous publication [22].

#### SH behaviors

Self-harm behaviors were defined as those outlined in the Modified version of the Adolescents Self-Harm Scale (MASHS), developed by Feng [23]. The participants were requested to answer 18 questions about the most commonly reported SH behaviors observed among Chinese adolescents, so as to evaluate the lifetime frequency and severity of SH.

#### Parenting styles

A Chinese version of the Short-Form of the Egna Minnen Barndoms Uppfostran (S-EMBU-C) was administered to assess parenting styles during childhood years [24]. This scale measures paternal and maternal parenting styles separately by using a uniform 21-item questionnaire. All items can be categorized into three dimensions: rejection, over-protection, and emotional warmth. The answer to each item was coded according to a four-point scale ranging from “never” (1 point) to “very often” (4 points). A higher combined score indicates a parenting style that was adopted more frequently. For our analytical sample, the Cronbach’ s alpha was 0.827 (Bootstrap 95% CI: 0.816–0.836).

#### Impulsivity

The Barratt Impulsiveness Scale (BIS) was used to evaluate impulsivity in participants. The BIS contains 30 items and three subscales, which assess impulsive planning, motor impulsiveness, and cognitive impulsiveness [25]. Each item can be scored from 1 to 5 based on a five-point scale. The total weighted score for the BIS ranges from 0 to 100, with a higher score indicating greater impulsivity. For our analytical sample, the Cronbach’ s alpha was 0.919 (Bootstrap 95% CI: 0.915–0.923).

### Statistical analysis

Descriptive analyses were used to delineate the participants’ distributional characteristics. Based on univariate analysis, multivariate logistic regression models were developed to measure the adjusted associations between parenting styles, impulsivity, and SH. The possible mediation via impulsivity was evaluated using path analysis. All statistical analyses were performed using R software (Version 3.6.2, The R Foundation for Statistical Computing, Vienna, Austria). In survey studies, especially those that employ a cluster sampling design, an intercorrelation between the participants may exist. Therefore, we utilized survey packages to control for this possible unequal sampling probability. For all analyses, the significance level was set as a two-tailed *p*-value less than 0.05.

## Results

### General features of study participants

Among all 3146 participants, 1480 reported SH behaviors, with a lifetime reported SH prevalence of 47.0% (95% CI: 36.3–58.0%); the median of the BIS was 41.67 (inter-quartiles range, IQR: 10.84); the median values for paternal parenting styles were 7 (rejection, IQR: 3), 15 (emotional warmth, IQR: 8), and 16 (over-protection, IQR: 4), whereas the median values for maternal parenting styles were 8 (rejection, IQR: 3), 14 (emotional warmth, IQR: 7), and 17 (over-protection, IQR: 4).

### SH behaviors, parenting styles, and impulsivity

For a better understanding of the results, we reversed the score of emotional warmth. Therefore, after reversion, a higher score indicated that an emotional warmth parenting style was adopted less frequently. The median of impulsivity (39.17) was used as the cut-off. After controlling for possible covariates, impulsivity was significantly associated with an increased occurrence of SH (OR = 2.07,95% CI: 1.75–2.44). For parenting styles, the results of the multivariate logistic regression models revealed the following: less emotional warmth from the father (OR = 1.05, 95% CI 1.02–1.09), maternal over-protection (OR = 1.08, 95% CI 1.02–1.14), and rejection (OR = 1.09, 95% CI 1.01–1.07) were associated with SH (Table 2).

**Table 2:** Univariate and multivariable Logistic regression models fitting results for SH

Covariates	Univariate	Mutilvariable1	Mutilvariable2	Mutilvariable3
	OR (95%CI)	OR (95%CI)	OR (95%CI)	OR (95%CI)
Age(+ 1 year)	1.27 (1.18, 1.37)	1.09 (0.90, 1.32)	1.12 (0.94, 1.33)	1.10 (0.91, 1.33)
Sex: Girls (Ref: Boys)	1.38 (1.24, 1.54)	1.44 (1.17, 1.78)	1.40 (1.13, 1.74)	1.42 (1.14, 1.76)
Grade (Ref: Primary school)				
Junior high school	2.38 (1.32, 4.31)	1.94 (0.86, 4.37)	1.90 (0.90, 4.02)	2.01 (0.88, 4.59)
Senior high school	3.47 (2.21, 5.45)	2.48 (0.94, 6.57)	2.27 (0.95, 5.42)	1.11 (1.00, 1.23)
Ethnicity (Ref: Other)	1.03 (0.81, 1.32)			
Father’s education level (Ref: Elementary and below)				
Junior high school above	0.79 (0.58, 1.07)			
Mother’s education level (Ref: Elementary and below)				
Junior high school above	0.70 (0.55, 0.90)	1.09 (0.97, 1.22)	1.10 (0.98, 1.23)	1.11 (1.00, 1.23)
Father’s age (+ 5 years)	1.18 (1.11, 1.25)	0.96 (0.87, 1.07)	0.95 (0.85, 1.06)	0.96 (0.86, 1.06)
Mother’s age (+ 5 years)	1.21 (1.10, 1.33)	1.07 (0.94, 1.22)	1.07 (0.93, 1.23)	1.09 (0.95, 1.26)
Impulsivity (Ref: Combined score < 39.17)				
High (Combined score > = 39.17)	3.34 (2.84–3.92)	2.16 (1.82, 2.55)	2.16 (1.82, 2.56)	2.07 (1.75, 2.44)
Parenting style				
Father’s Rejection (Combined score + 1)	1.20 (1.14–1.26)	1.13 (1.07, 1.20)		1.06 (0.97, 1.15)
Father’s Emotional Warmth (Combined score + 1)	1.08 (1.06–1.10)	1.05 (1.03, 1.07)		1.05 (1.02, 1.09)
Father’s Over-protection (Combined score+ 1)	1.12 (1.10–1.16)	1.07 (1.03, 1.11)		1.01 (0.93, 1.09)
Mother’s Rejection (Combined score + 1)	1.22 (1.17–1.26)		1.13 (1.08, 1.19)	1.09 (1.01, 1.17)
Mother’s Emotional Warmth (Combined score + 1)	1.08 (1.07–1.09)		1.05 (1.02, 1.07)	1.00 (0.96, 1.03)
Mother’s Over-protection (Combined score + 1)	1.13 (1.11–1.15)		1.08 (1.05, 1.11)	1.08 (1.02, 1.14)

### Path analysis

**Fig. 1:**
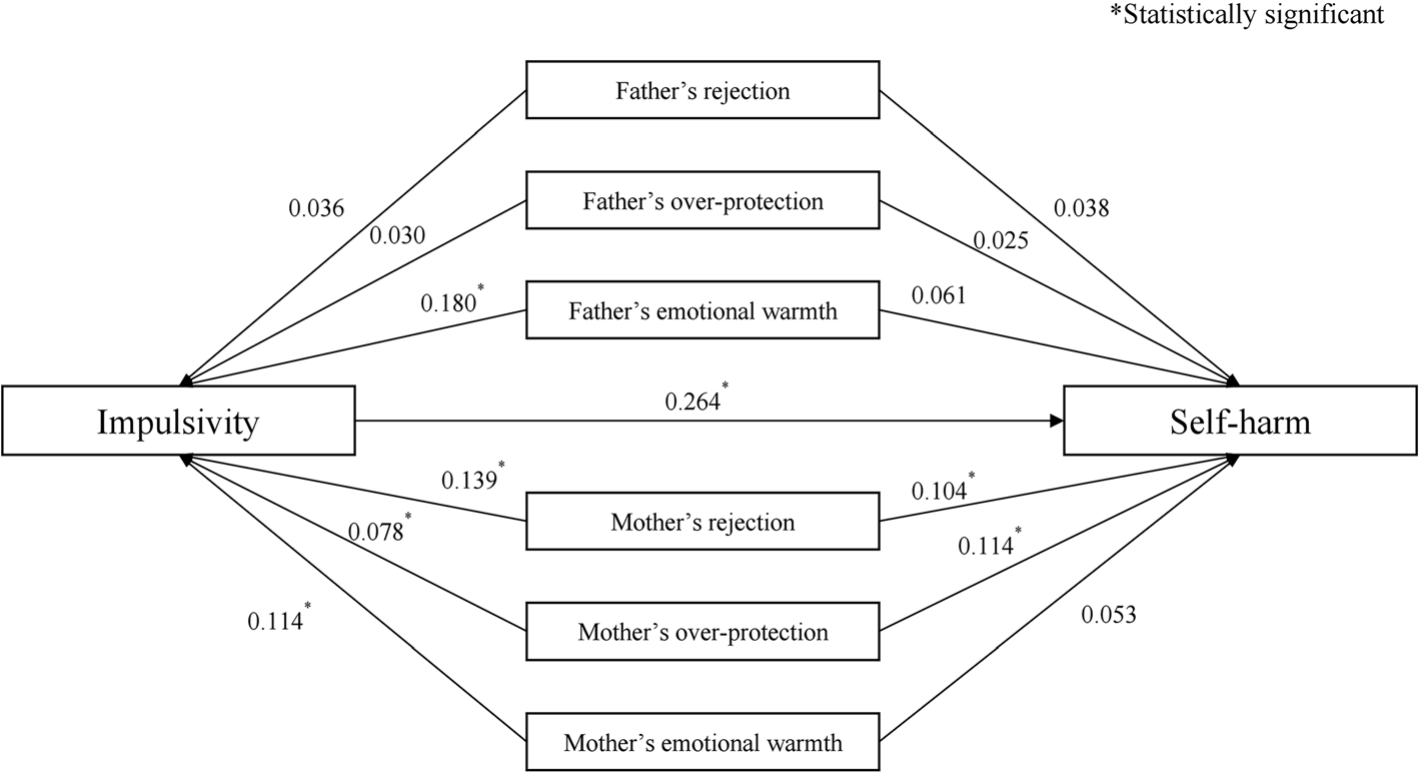
Path model for father and mother’s parenting-SH. Impulsivity and SH were adjusted for: age, sex and grade

Based on the results of the multivariate logistic regression models, we constructed an initial hypothesized path framework for paternal and maternal parenting styles. This path model achieved ideal fitting: The Root Mean Square Error of Approximation (RMSEA) was 0.001, and the Goodness-of-Fit Index (GFI) was 0.998. As we expected, impulsivity played a mediating role, as suggested by the model. However, only two paths (maternal rejection and over-protection) were statistically significant. Based on the standardized correlation coefficients shown in Fig. 1, the direct association between parenting styles and SH was 0.218, and the indirect association mediated via impulsivity was 0.067, accounting for 23.43% of the total association (Fig. 1). Further details regarding the bootstrap CIs for the indirect paths from parenting styles to SH are provided in the supplementary materials (Table S1).

**Fig. 2:**
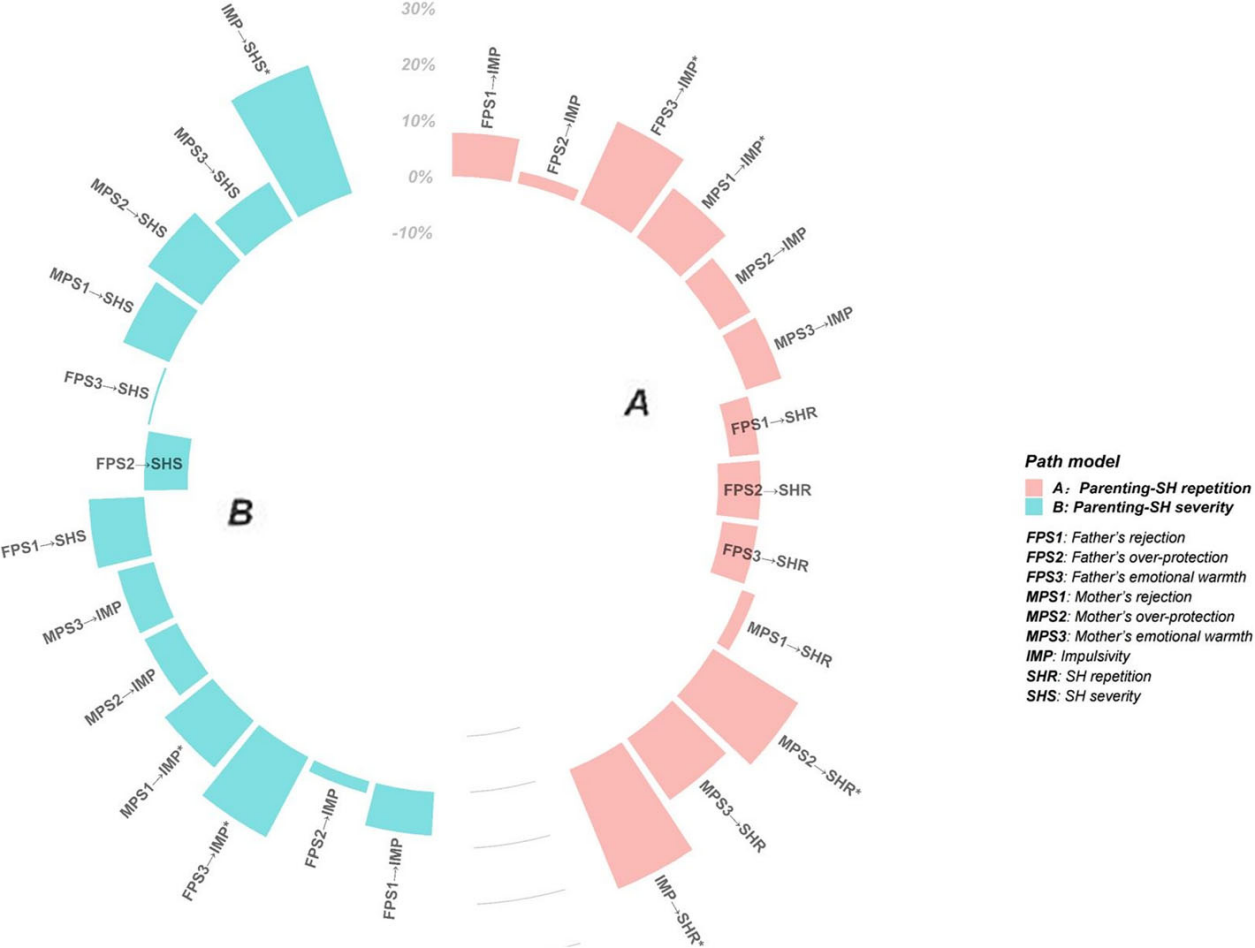
Mediation of impulsivity in the associations between parenting styles and SH repetition, SH severity. FPS1: Father’s rejection; FPS2: Father’s over-protection; FPS3: Father’s emotional warmth; MPS1: Mother’s rejection; MPS2: Mother’s over-protection; MPS3: Mother’s emotional warmth; IMP: Impulsivity; SHR: SH repetition; SHS: SH severity

Among individuals who self-harm, a significant association is observed between suicidal behaviors and the repetition and severity of SH. A such, we performed an additional subgroup analysis in 1480 SH adolescents, in an effort to explore impulsivity as a mediator in the association between parenting styles and SH repetition and SH severity. However, only non-significant associations were observed (Fig. 2).

## Discussion

In the current study, based on the results from a large sample population of Chinese children and adolescents, we found that the prevalence of lifetime SH was 47.0%, which was much higher than a pooled estimation of 22.4% among Chinese adolescents involved in the aforementioned meta-analysis [5]. The utilization of different SH instruments may largely explain this discrepancy: In the current study, we used MASHS to measure lifetime SH in the participants, whereas in the meta-analysis, most of the studies included applied short answer questions (SAQ) to evaluate SH behavior over a period of 6 months or 12 months. Moreover, the age distribution of the study participants differed. The current study included children and adolescents aged between 10 and 17 years old, whereas all of the participants in the meta-analysis were middle school and high school students.

Based on the results of the analysis, we found that less emotional warmth from the father, maternal over-protection, and rejection were significantly associated with SH. This conclusion was in line with two previous studies, which suggested that adverse parenting styles were risk factors of SH and suicidal behaviors among a representative sample of 3653 Chinese adolescents [12, 26]. Parental over-protection and rejection can take the form of a stricter attitude and a higher level of control, and they have a negative effect on a child’s confidence and independence, which may in turn lead to mental health issues [27]. For instance, a higher level of parental control could predict depression and anxiety among adolescents [28, 29]. Due to cultural differences, Chinese parenting styles are distinct from those of western countries: Chinese parents, especially mothers, are more inclined to adopt over-protective parenting styles, which derive from traditional ideology, such as Confucianism [30]. A classic model of harsh maternal parenting in China is that of the “tiger mother”, which generally describes mothers who exercise excessive control and harsh discipline towards their children [31]. A longitudinal study revealed that tiger parenting is associated with high academic pressure, depression, and a sense of alienation, all of which were identified as contributing factors in SH in youngsters [32]. Impulsivity, as a mediator of SH, was only observed in the case of maternal over-protection. Therefore, with respect to adolescents who reported experiences of a harsh maternal parenting style, it is reasonable to suggest that intervention measures aimed at reducing impulsivity may achieve a better effect in the area of SH prevention and control. Another reasonable explanation concerns the way in which strict and harsh parenting, such as over-protection and rejection, may hinder the development of proper impulse controls in adolescents: Longitudinal evidence found higher levels of reported impulsivity among young people who experienced parental coercion [33].

After controlling for potential confounding factors, the hypothesized path model achieved ideal compatibility with our data, and it suggested that impulsivity was a meaningful mediator in the association between parenting styles and SH. Impulsivity is widely associated with many adolescent risk behaviors, especially SH [14]. Existing studies showed that adolescents who had engaged in SH generally reported a higher impulsivity level [34]. A longitudinal study revealed that the association between impulsivity and SH may be reciprocal, such that a higher level of impulsivity may lead to an increased risk of SH, whereas more frequent SH behaviors may exacerbate tendencies towards impulsivity [14]. Some established models of impulsivity, such as the UPPS-P model and the Barratt model, suggested that individuals who reported SH were more inclined to act impulsively when experiencing negative emotions or events [14, 35–37]. Another possible theory is that impulsivity may increase the likelihood of SH behavior by reinforcing the processes involved in SH ideation or thought: In their previous study, Madge et al. identified a series of prominent dose-response associations between cognitive impulsivity and SH ideation, SH occurrence, and SH events [38].

Impulsivity, as a personality trait, is closely associated with parenting styles. Parenting styles influence a child’s development in the form of behaviors and personalities. Adverse parenting styles may lead to early maladaptive schemas (EMS) which begin to form in early childhood. Such schemas may have a long-lasting adverse effect that persists into adulthood, contributing to the development of affective and personality disorders [10, 39–41]. Shu et al. reported that parental rearing patterns, particularly rejection and over-protection, were predictors of an impulsive personality [18]. Considering all of the above, it is reasonable to identify impulsivity as a significant mediator in the association between parenting styles and SH. With the exception of direct interventions aimed at nurturing positive parenting styles, this likely mediation factor also highlights the need to design and implement impulsivity-centered intervention measures, in an effort to curb parenting styles that contribute to the incidence of SH in adolescents. In fact, some promising behavior management procedures, such as the Good Behavior Game (GBG), could be incorporated into coping strategies that are designed to manage impulsivity or other disruptive behaviors [42]. Nevertheless, the performance of the GBG should be meticulously evaluated before implementation.

Some limitations of the current study should be noted. First, this study employed a cross-sectional design, which means that it is not possible to make causal inferences. Moreover, the sample in this study included participants from a locality in southwest China. Therefore, the generalization of the results to the wider Chinese adolescent population should be made with caution. Future studies should be carried out among a larger and more representative Chinese adolescent population. Finally, we relied on the self-report method to collect the survey data. As such, it is possible that the data may be affected by information bias. Studies which utilize multiple reporting sources (such as parents and relatives) should be considered.

Despite all of these limitations, our study adopted a novel approach to investigating impulsivity as a mediator in the association between parenting styles and SH among Chinese adolescents. Our major findings can contribute to furthering an understanding of the complicated relationship between parenting styles and SH. Future studies with prospective designs are needed to corroborate our major findings.

## Conclusion

This population-based study employed a cross-sectional design to examine parenting styles including less paternal emotional warmth, maternal over-protection, and rejection, which were significantly associated with SH, and impulsivity was identified as a clear mediator in the association between parenting styles and SH. Our major findings suggest that, for Chinese adolescents who experience a harsher maternal parenting style, impulsivity-centered intervention measures might be effective in reducing parenting-style-related SH.

## Supplementary Information

**Additional file 1: Table S1.** Bootstrap confidence intervals for indirect paths from parenting styles to SH.

## Data Availability

The datasets analyzed during the current study are available from the corresponding author upon reasonable request.

## Abbreviations

SH: Self-harm
MASHS: The Modified Version of Adolescents Self-Harm Scale
S-EMBU-C: The Chinese Version of the Short-Egna Minnen Barndoms Uppfostran
BIS: Barratt Impulsiveness Scale
CI: Confidence interval
SE: Standard error
IQR: Interquartile range
OR: Odds ratio
RMSEA: Root mean square error of approximation
GFI: Goodness-of-fit index
