# Supplementary material for: Impulsivity mediates the association between parenting styles and self-harm in Chinese adolescents
Source: BMC Public Health. 2021 Feb 10;21:332. doi: 10.1186/s12889-021-10386-8 (PMC7877034; doi:10.1186/s12889-021-10386-8)
Supplement: Supplementary file 1 — Additional file 1: Table S1. Bootstrap confidence intervals for indirect paths from parenting styles to SH. [file 12889_2021_10386_MOESM1_ESM.docx]

**Table S1** Bootstrap confidence intervals for indirect paths from parenting styles to SH

| Parenting style | Indirect path | Estimation | 95% bootstrap CI |
| --- | --- | --- | --- |
| Father | Rejection → impulsivity | 0.147 | (-0.092,0.387) |
|  | Over protection → impulsivity | 0.071 | (-0.083,0.225) |
|  | Emotion warmth → impulsivity | 0.281 | (0.158,0.404) |
| Mother | Rejection → impulsivity | 0.537 | (0.307,0.768) |
|  | Over protection → impulsivity | 0.179 | (0.030,0.328) |
|  | Emotion warmth → impulsivity | 0.184 | (0.056,0.313) |
